# Comprehensive somatosensory and neurological phenotyping of NCS1 knockout mice

**DOI:** 10.1038/s41598-021-81650-5

**Published:** 2021-01-27

**Authors:** Lien D. Nguyen, Luca G. Nolte, Winston J. T. Tan, Denise Giuvelis, Joseph Santos-Sacchi, Edward Bilsky, Barbara E. Ehrlich

**Affiliations:** 1 Department of Pharmacology, Yale University, 333 Cedar St, New Haven, CT USA; 2 Interdepartmental Neuroscience Program, Yale University, New Haven, CT USA; 3 Department of Surgery (Otolaryngology), Yale University, New Haven, CT USA; 4 Center for Excellence in the Neurosciences, University of New England, Biddeford, ME USA; 5 Department of Cellular and Molecular Physiology, Yale University, New Haven, CT USA; 6 Department of Neuroscience, Yale University, New Haven, CT USA; 7 Department of Biomedical Sciences, Pacific Northwest University of Health Sciences, Yakima, WA USA; 8 Present Address: Department of Neurology, Ann Romney Center for Neurologic Diseases, Brigham and Women’s Hospital and Harvard Medical School, Boston, MA USA; 9 Present Address: Charité – Universitätsmedizin Berlin, Corporate Member of Freie Universität Berlin, Humboldt-Universität Zu Berlin, and Berlin Institute of Health, Klinik Und Hochschulambulanz Für Neurologie, 10117 Berlin, Germany

**Keywords:** Neuroscience, Auditory system, Molecular neuroscience, Sensory processing

## Abstract

Neuronal calcium sensor 1 (NCS1) regulates a wide range of cellular functions throughout the mammalian nervous systems. Altered NCS1 expression is associated with neurodevelopmental and neurodegenerative diseases. Previous studies focused on affective and cognitive behaviors in NCS1 knockout (KO) mice, but little is known about the physiological and pathological states associated with the loss of NCS1 in the peripheral nervous system. We previously reported that NCS1 expression was reduced following paclitaxel-induced peripheral neuropathy. Here, we comprehensively investigated the phenotypes of NCS1-KO mice through a battery of behavioral tests examining both central and peripheral nervous systems. Generally, only mild differences were observed in thermal sensation and memory acquisition between NCS1-WT and -KO male mice, but not in female mice. No differences were observed in motor performance, affective behaviors, and hearing in both sexes. These results suggest that NCS1 plays a modulatory role in sensory perceptions and cognition, particularly in male mice. NCS1 has been proposed as a pharmacological target for various diseases. Therefore, the sex-specific effects of NCS1 loss may be of clinical interest. As we examined a constitutive KO model, future studies focusing on various conditional KO models will further elucidate the precise physiological significance of NCS1.

## Introduction

Calcium (Ca^2+^) is a crucial secondary messenger that regulates a wide range of cellular functions, including metabolism, motility, and secretion^1^. In the nervous system, Ca^2+^ regulates exocytosis during synaptic transmission, long-term potentiation and depression, and spine and dendrite formation – crucial processes required for memory consolidation, learning, and other higher cognitive functions^2^. Consequently, disrupted Ca^2+^ signaling has been associated with neurodevelopmental and neurodegenerative diseases^3^. To fulfill all those distinct, subtle, and diverse functions, Ca^2+^ acts through binding partners such as Ca^2+^ sensor proteins. Examples of families of calcium-binding proteins include the neuronal calcium sensors, the stromal interaction molecule (STIM) proteins, and S100 proteins^4^. Aberrant expression of these interacting partners may result in neurological defects. For example, both heterozygous mice deficient in Ca^2+^/calmodulin-dependent protein kinase IIα (CaMKIIα)^5^ and mice overexpressing S100β exhibited learning and memory impairment^6^.

Here, we focus on neuronal calcium sensor 1 (NCS1), a highly conserved member of the neuronal calcium sensor family with three functional EF-hand domains. Several studies have shown that NCS1 plays an essential role in neurotransmission^7^, neurite outgrowth^8^, short- and long-term potentiation^9^, as well as neuronal survival^10^. Aberrant expression or function of NCS1 has been implicated in the pathophysiology of diseases such as bipolar disorder and schizophrenia^11^, cocaine addiction^12^, Parkinson's disease^13^, autism^14,15^, and fragile X syndrome^16^. We also previously described that paclitaxel, a commonly used chemotherapeutic in the treatment of solid tumors, binds NCS1 and enhances Ca^2+^ influx to the cytosol, which in turn activates calpain. Calpain degrades NCS1 and several other proteins, which leads to the development of peripheral neuropathy^17–19^. These findings suggest that NCS1 deficiency may adversely affect the peripheral nervous system, leading to symptoms of peripheral neuropathies affecting sensory and motor systems. Therefore, we aimed to investigate if NCS1-KO mice also showed deficits in sensory and motor systems. Furthermore, the majority of published research only utilized male mice, which may neglect sex-specific differences. It has been well-established that male and female mice behave differently^20–22^. Differences between sexes were also observed in pain perception^23^. As the over-reliance on male animals may erroneously inform clinical studies^24^, we examined both sexes in our study.

Here, we investigated the effects of the loss of NCS1 in the peripheral and central nervous systems in both male and female mice through assessments of sensory, motor, and cognitive behaviors. We found mild differences in sensory and motor behaviors affecting male NCS1-KO mice specifically, whereas female NCS1-KO mice were largely comparable to female NCS1-WT mice. Our results suggest a sex- and modality-specific role of NCS1 in behavioral functions. As other groups and we have proposed NCS1 as a pharmacological target for treating various neurological diseases, particularly neurodevelopmental and psychiatric diseases^16,19,25–30^, future studies will also benefit from considering potential sex-specific differences in NCS1 functions.

## Results

### Sensory behaviors

#### Male NCS1-KO mice show higher sensitivity to 50 °C thermal stimulus

**Figure 1:**
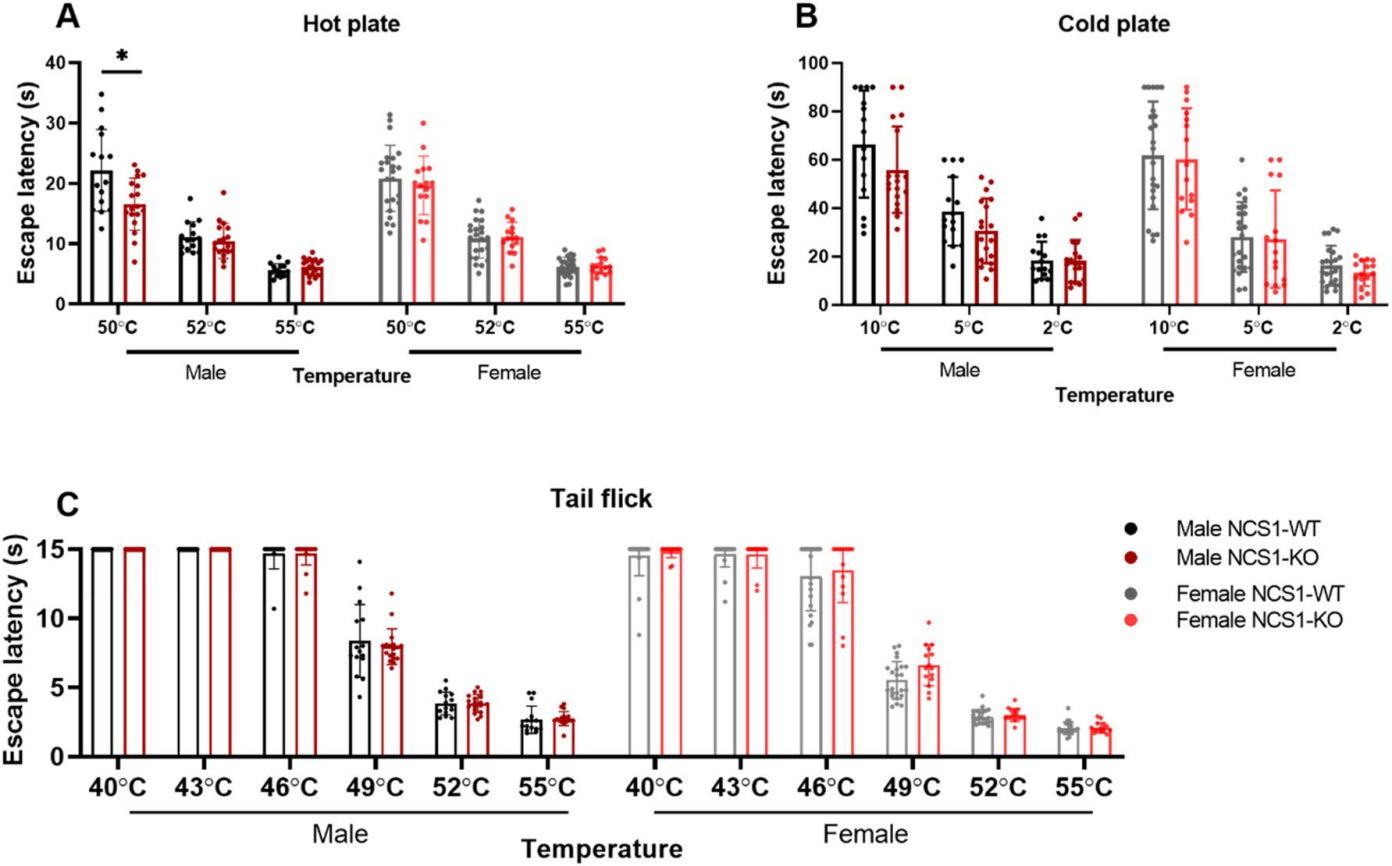
NCS1-WT and -KO mice exhibit mostly similar thermal sensation. (**A**) Compared to male NCS1-WT mice, male NCS1-KO mice displayed a shorter escape latency in a hot plate test only at 50 °C (2-way ANOVA, Factor genotype, *p* = 0.03, followed by multiple comparison test with Sidak’s correction, temperature 50° C, *p* = 0.03). No differences were observed between female NCS1-WT and NCS1-KO mice (2-way ANOVA, Factor genotype, *p* = 0.75). No differences were found at any temperature between NCS1-WT and NCS1-KO mice in the (**B**) cold plate test [2-way ANOVA (male), Factor genotype, *p* = 0.13; 2-way ANOVA (female), Factor genotype, *p* = 0.66] and (**C**) tail flick test [2-way ANOVA (male), Factor genotype, *p* = 0.81; 2-way ANOVA (female), Factor genotype, *p* = 0.21]. Error bars show SD. Male NCS1-WT: n = 15, male NCS1-KO: n = 18, female NCS1-WT: n = 15, female NCS1-KO: n = 23.

To assess the peripheral nervous system, basal temperature response latencies were measured using the hot plate test, the cold plate test, and the warm water tail-immersion test (Fig. 1). For the hot plate test, male NCS1-KO mice exhibited shorter latencies to escape at 50 °C (Fig. 1A, 2-way ANOVA, followed by multiple comparison test with Sidak’s correction, *p* = 0.03), suggesting a higher sensitivity to the 50 °C thermal stimulus compared to male NCS1-WT mice. No differences in latencies were observed at 52 °C and 55 °C. Female NCS1-KO and NCS1-WT performed similarly in the hot plate test across all three temperatures examined. No differences were found at any temperature between NCS1-WT and NCS1-KO mice in the cold plate test (Fig. 1B) and tail-flick test (Fig. 1C). In all three tests, mice in all groups showed a decrease in escape latency when the test temperature became more intense (2-way ANOVA, factor temperature, *p* < 0.0001), suggesting that the tests accurately detected avoidance behaviors towards increasingly intense noxious thermal stimuli.

**Figure 2:**
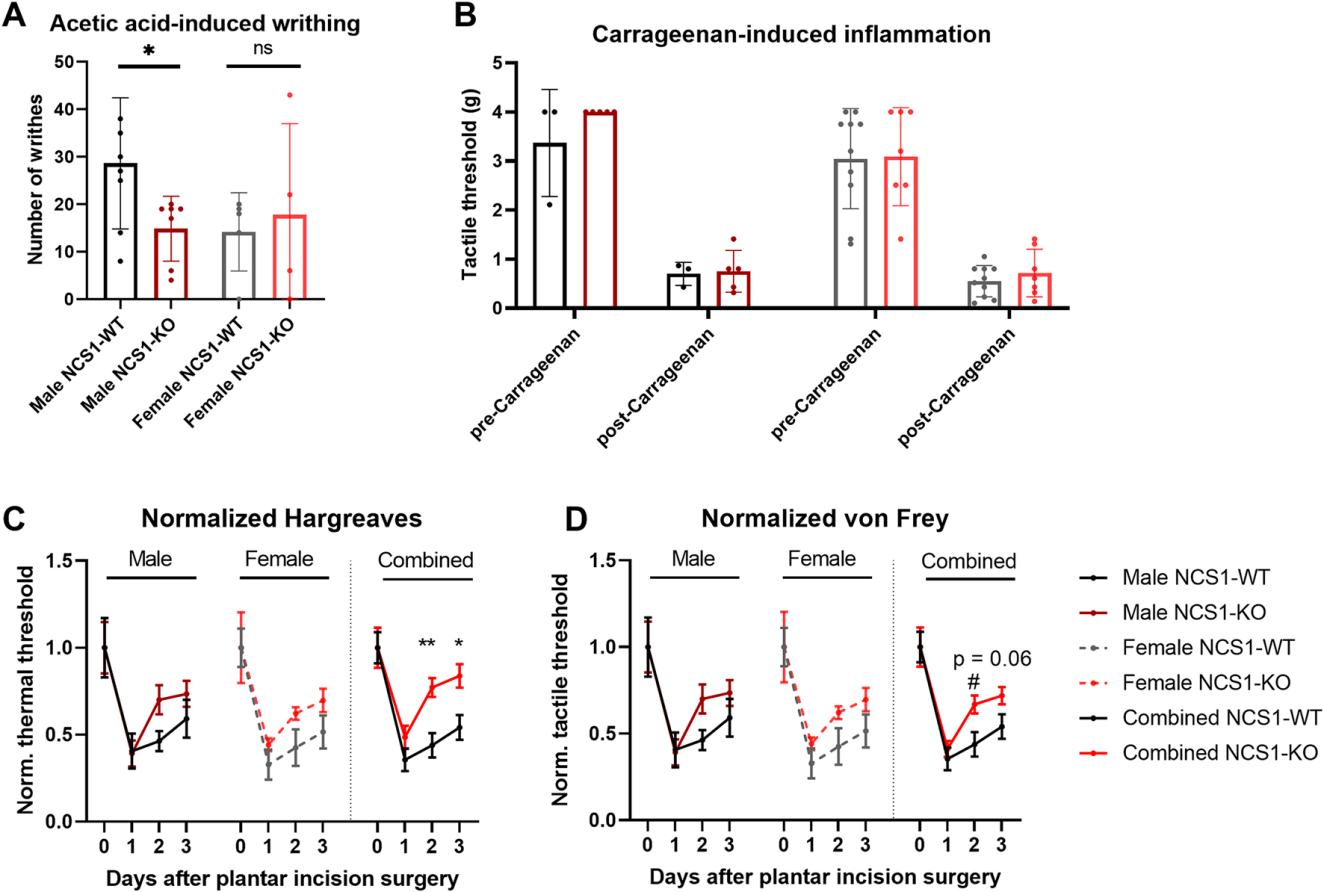
NCS1-WT and -KO mice exhibit differences in pain sensation. (**A**) Male NCS1-KO mice (n = 7) exhibited fewer writhes compared to -WT controls (n = 8) after dilute acetic-acid injection (unpaired t-test, *p* = 0.03), whereas female mice (NCS1-KO: n = 4, NCS1-WT: n = 5) showed no difference between genotypes (unpaired t-test, *p* = 0.7176). (**B**) No differences were observed between NCS1-KO (male NCS1-KO: n = 5, female NCS1-KO: n = 7) and -WT mice (male NCS1-WT: n = 3, female NCS1-WT: n = 10) in the Carrageenan inflammatory pain assay [2-way ANOVA (male), Factor genotype, *p* = 0.20; 2-way ANOVA (female), Factor genotype, *p* = 0.73]. (**C**) The normalized Hargraves assay shows differences between NCS1-KO (female NCS1-KO: n = 4, male NCS1-KO: n = 6) and -WT mice (female NCS1-WT: n = 8, male NCS1-WT: n = 4) 2 and 3 days after plantar incision surgery (2-way ANOVA, Factor genotype, *p* = 0.03, followed by multiple comparison test with Sidak’s correction, day 2, *p* = 0.01, day 3, *p* = 0.02). (**D**) The normalized von Frey assay indicates a difference between NCS1-KO (male NCS1-KO: n = 6, female NCS1-KO: n = 4) and -WT (male NCS1-WT: n = 8, female NCS1-KO: n = 4) 2 days after plantar incision surgery (2-way ANOVA, Factor genotype, *p* = 0.16, followed by multiple comparison test with Sidak’s correction, day 2, *p* = 0.06). Line graphs show mean ± SEM, bar graphs show mean ± SD.

#### Male NCS1-KO mice show fewer acetic acid-induced writhes

Chemical nociception of visceral (subdiaphragmatic organs) and subcutaneous organs (muscle wall) was assessed using the acetic-acid writhing assay (Fig. 2). Male NCS1-WT mice exhibited a robust writhing response to the peritoneal injection of dilute acetic acid, whereas NCS1-KO mice had significantly fewer writhes under the same conditions (Fig. 2A, unpaired two-tailed t-test, *p* = 0.03). On average, female NCS1-WT and NCS1-KO mice had a less intense writhing response than male mice but showed no difference between the genotype (*p* > 0.05). Tactile sensory thresholds were assessed before and after carrageenan injection into the plantar surface of the mouse's left hind paw, a model of inflammatory-induced hyperalgesia. As expected, both male and female NCS1-WT groups demonstrated a decrease in tactile threshold (allodynia) following the carrageenan administration as assessed by von Frey filaments (Fig. 2B, 2-way ANOVA, Factor time, *p* < 0.0001). NCS1-KO mice exhibited a similar change in threshold compared to NCS1-WT controls (Fig. 2B).

#### NCS1-KO mice exhibit faster recovery from tactile allodynia

Changes in mechanical and thermal sensory thresholds in response to a standard rodent model of post-surgical pain (hind paw incision) were examined before, and then 1, 2, and 3 days post-injury. As expected, the injury model produced both tactile allodynia and thermal hyperalgesia that peaked in intensity on post-injury day 1 (2-way ANOVA, Factor time, *p* < 0.001). No difference in tactile threshold (von Frey) was observed between NCS1-WT and NCS1-KO mice when analyzing male and female mice separately, although the sample size was small (n = 4 for each sex). When both males and females were combined for analysis of recovery times, NCS1-KO mice showed a more rapid return to pre-injury baseline thresholds on days 2 and 3 compared to NCS1-WT controls (Fig. 2C, 2-way ANOVA, Factor genotype, *p* = 0.03, followed by multiple comparison test with Sidak’s correction, day 2: *p* = 0.01, day 3: *p* = 0.02). Thermal hyperalgesia was assessed using a Hargreaves apparatus^31^ with the same timeline as mechanical thresholds (baseline and 1–3 days post-injury). No sex differences were observed, and when combined, NCS1-KO mice also showed a trend towards faster recovery compared to NCS1-WT controls (Fig. 2D, day 2: *p* = 0.06).

#### NCS1-WT and -KO mice exhibit similar peripheral hearing

**Figure 3:**
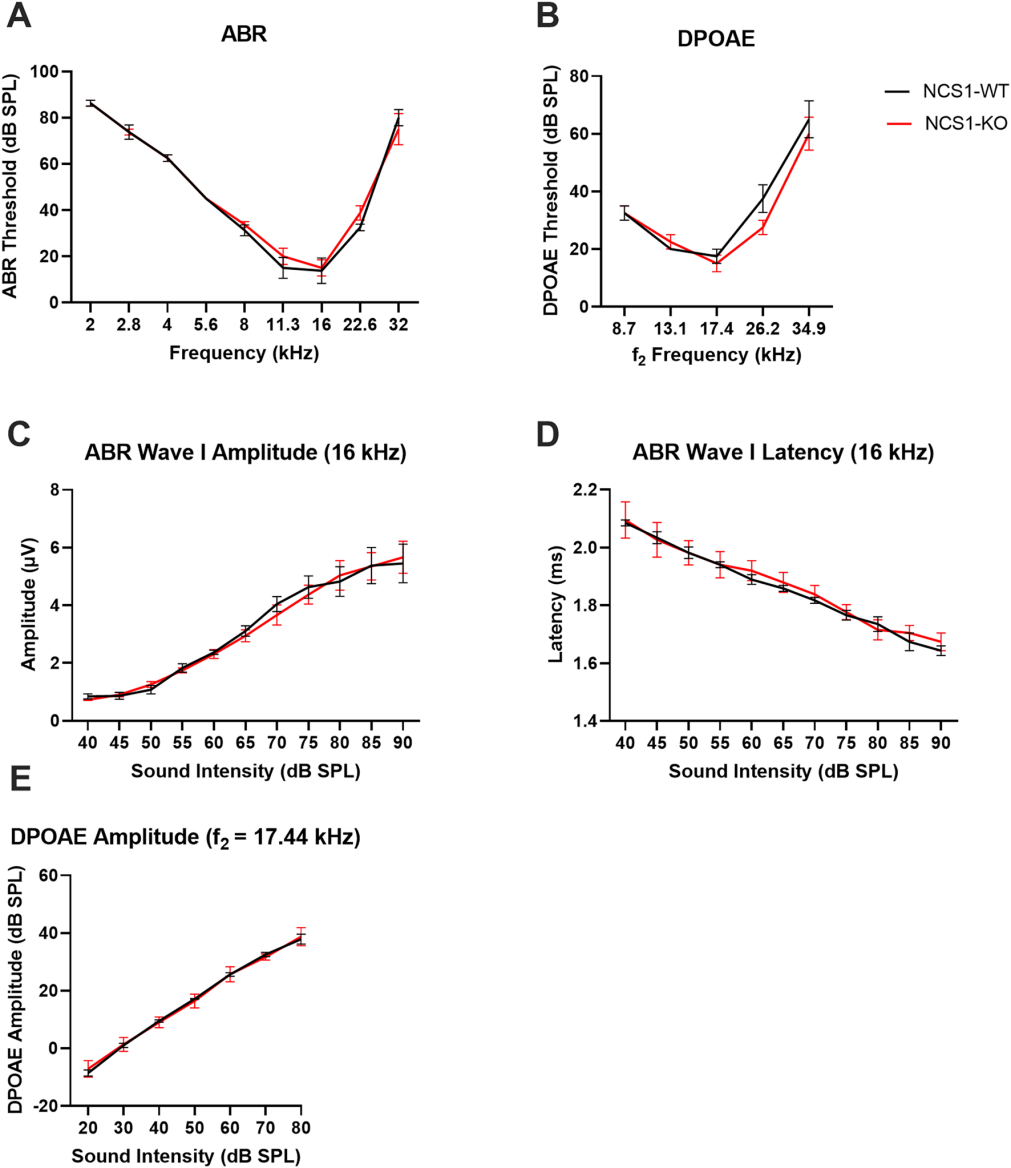
NCS1-WT and -KO mice exhibit similar hearing. (**A**,**B**) The graphs illustrated the average ABR (**A**) and DPOAE (**B**) thresholds of NCS1-KO and -WT mice as a function of stimulus frequency. There were no significant differences in both ABR (2-way ANOVA, Factor genotype, *p* = 0.67) and DPOAE (2-way ANOVA, Factor genotype, *p* = 0.22) thresholds between NCS1-KO and NCS1-WT mice across the frequency range tested. (**C**,**D**) The graphs illustrated the average amplitude (**C**) and latency (**D**) of ABR wave I as a function of sound intensity at 16 kHz. There were no significant differences in both amplitudes and latencies between NCS1-KO and NCS1-WT mice at all sound intensities. (**E**) The graph illustrated the average DPOAE amplitude as a function of sound intensity at the f_2_ frequency of 17.44 kHz. There were no significant differences in DPOAE amplitudes at all sound intensities. Error bars are ± SEM. Male NCS1-WT: n = 2, male NCS1-KO: n = 2, female NCS1-WT: n = 2, female NCS1-KO: n = 2.

Hearing function was assessed using the auditory brainstem response (ABR) and distortion product otoacoustic emission (DPOAE) tests. ABRs measure evoked potentials from the auditory nerve and ascending auditory pathways in response to acoustic stimuli to assess cochlea function. No differences in ABR thresholds were observed between NCS1-WT and age-matched NCS1-KO mice across the frequency range tested (Fig. 3A, 2-way ANOVA, Factor genotype, *p* = 0.67, Factor frequency, *p* < 0.0001). DPOAEs measure sounds generated by outer hair cells (OHCs) in the cochlea in response to a two-tone stimulus to assess the robustness of OHC function. No differences in DPOAE thresholds were observed between NCS1-WT and age-matched NCS1-KO mice across the frequency range tested (Fig. 3B, 2-way ANOVA, Factor genotype, *p* = 0.22, Factor frequency, *p* < 0.0001). In addition, there were no differences in DPOAE amplitudes at all sound intensities (Fig. 3E). These results suggest that cochlea and OHC functions are unaffected by the loss of NCS1. Because a recent study^32^ demonstrated expression of NCS1 in type I spiral ganglion neurons, we further analyzed ABR wave I, which represents the activity of the auditory nerve, to determine if auditory nerve function was affected in NCS1-KO mice. Our results showed no significant differences in both wave I amplitudes, which reflect the number of activated neurons and synchrony of firing, and latencies, which reflect the timing of synaptic transmission and nerve conduction, between NCS1-WT and NCS1-KO mice (Fig. 3C, D). This suggests that the auditory nerve and the inner hair cells (IHCs) to which the auditory nerve innervates are unimpaired by the loss of NCS1.

### Motor behaviors

#### NCS1-WT and -KO mice show similar performance on motor coordination and grip strength tasks examined

**Figure 4:**
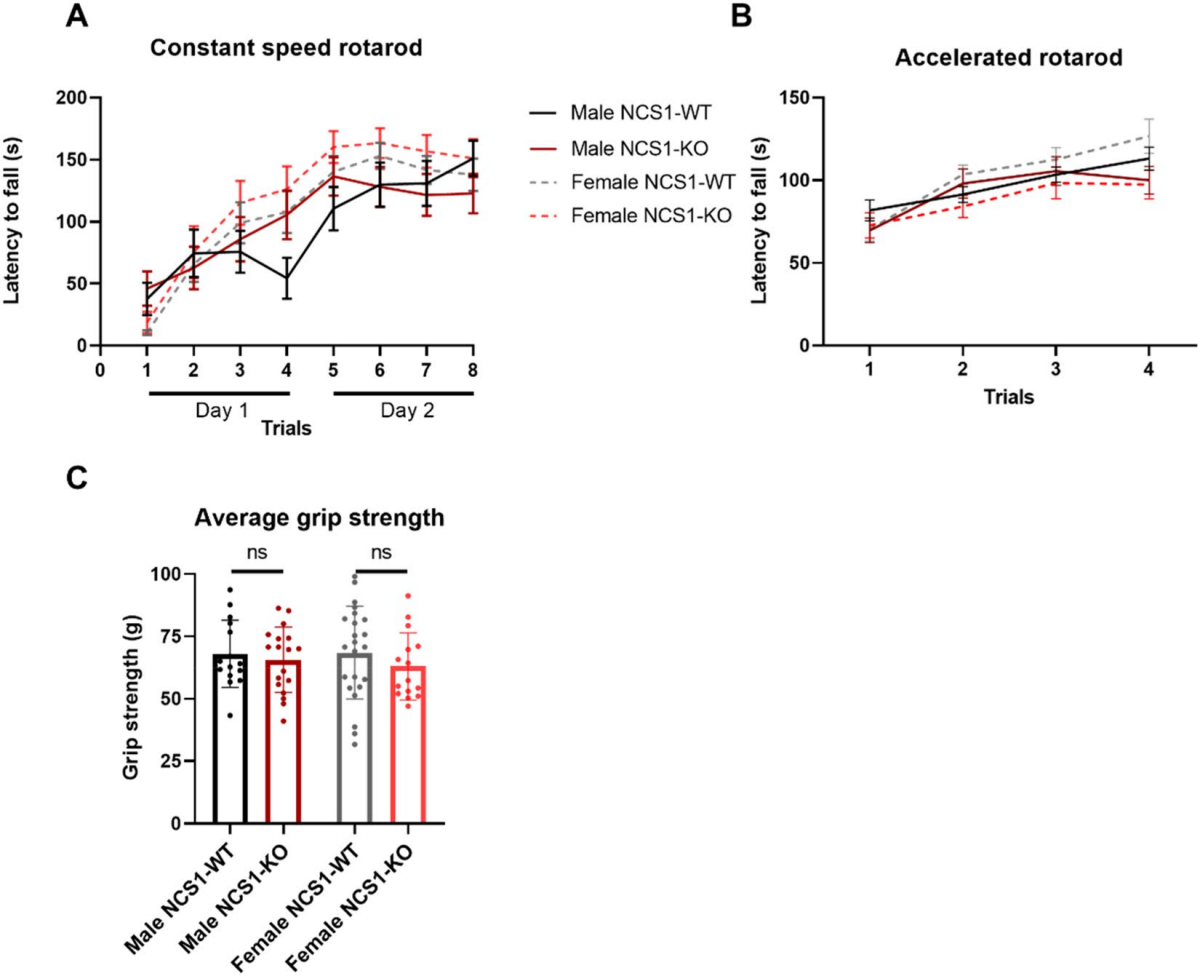
NCS1-WT and -KO mice perform similarly on motor tasks. (**A**) Although mice in all groups improved over time in the constant speed rotarod task [2-way ANOVA (male), Factor trial, *p* < 0.0001; 2-way ANOVA (female), Factor time, *p* < 0.0001], no differences were found between groups [2-way ANOVA (male), Factor genotype, *p* = 0.73; 2-way ANOVA (female), Factor genotype, *p* = 0.31]. (**B**) Similar results were found in the accelerated rotarod test [2-way ANOVA (male), factor genotype, *p* = 0.57; 2-way ANOVA (female), Factor genotype, *p* = 0.07]. (**C**) No differences were found in grip strength between male NCS1-WT and NCS1-KO mice (unpaired t-test, *p* = 0.61), or between female NCS1-WT and NCS1-KO mice (unpaired t-test, *p* = 0.33). Line graphs show mean ± SEM, bar graphs show mean ± SD For constant speed rotarod and grip strength. For grip strength and constant speed rotarod, male NCS1-WT: n = 15, male NCS1-KO: n = 18, female NCS1-WT: n = 15, female NCS1-KO: n = 23. For accelerated rotarod, male NCS1-WT: n = 13, male NCS1-KO: n = 13, female NCS1-WT: n = 9, female NCS1-KO: n = 10.

We next examined whether NCS1-KO mice showed differences in motor learning abilities using a constant speed rotarod test. Neither female nor male mice showed differences in performance depending on their genotype (Fig. 4A). Both genders showed better performance over time, suggesting motor learning (2-way ANOVA, Factor trial, *p* < 0.0001). The accelerated rotarod also showed improvement over time, but there were no differences between the genotypes (Fig. 4B). Using the grip strength task to examine neuromuscular functions (Fig. 4C), we also found no differences comparing male NCS1-WT and NCS1-KO mice, or between female NCS1-WT and NCS1-KO mice. These results suggest that the absence of NCS1 does not affect motor strength, motor coordination, or learning under the conditions examined.

#### Male NCS1-KO mice show slight hyperactivity in the open-field exploration task (OFE)

**Figure 5:**
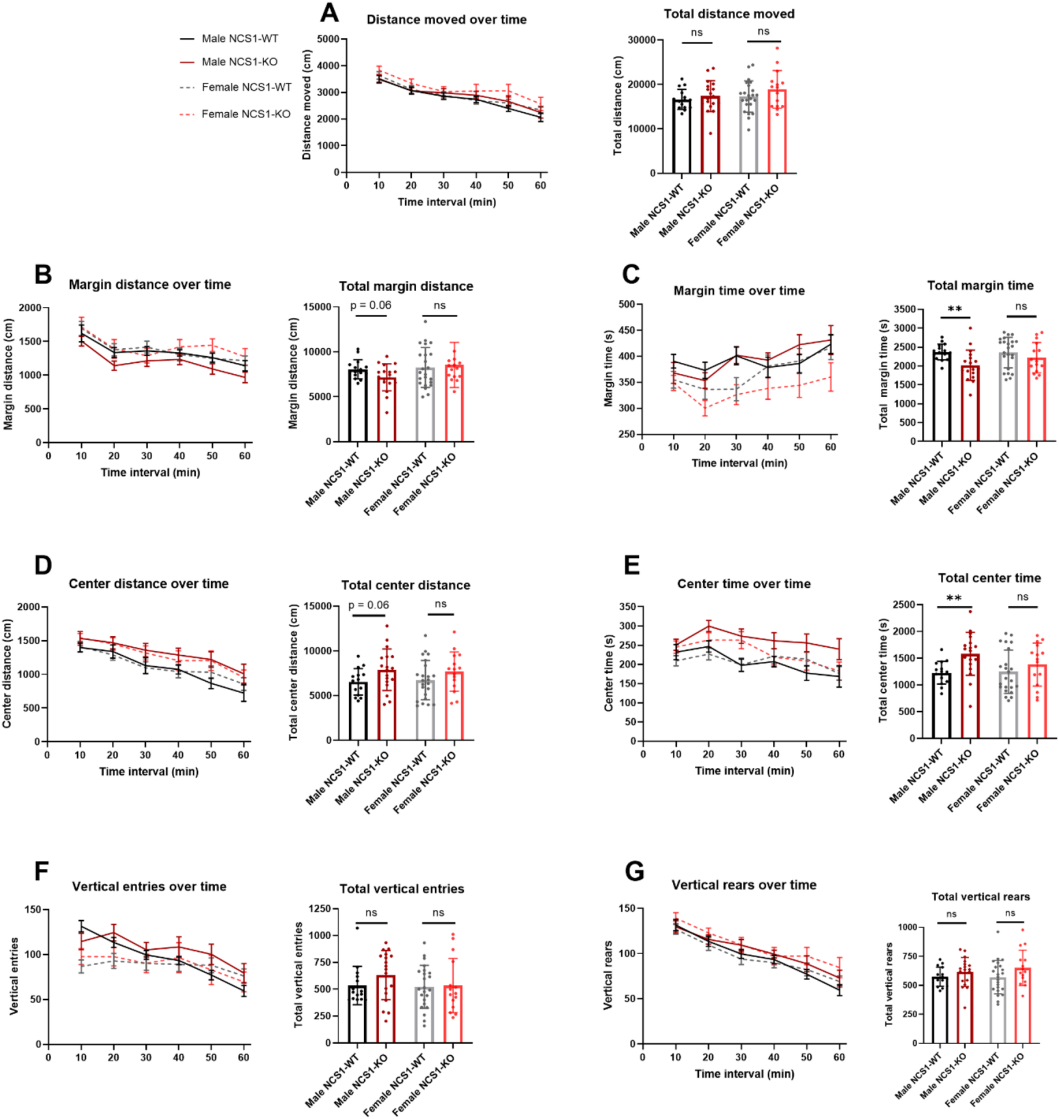
Male NCS1-KO mice exhibit hyperactivity in the open-field exploration task. Graphs show (**A**) distance moved (no differences), (**B**) margin distance (unpaired t-test, *p* = 0.06 for male NCS1-WT vs. male NCS1-KO, (**C**) margin time (unpaired t-test, *p* = 0.005 for male NCS1-WT vs. male NCS1-KO), (**D**) center distance (unpaired t-test, *p* = 0.06 for male NCS1-WT vs. male NCS1-KO), (**E**) center time (unpaired t-test, *p* = 0.005 for male NCS1-WT vs. male NCS1-KO); (**F**) vertical entries (no differences), and (G) vertical rears (no differences). Line graphs show mean ± SEM. Bar graphs show mean ± SD. Two-tailed student t-test was used to compare male NCS1-WT with male NCS1-KO, and female NCS1-WT with female NCS1-KO. Male NCS1-WT: n = 15, male NCS1-KO: n = 18, female NCS1-WT: n = 15, female NCS1-KO: n = 23.

We sought to examine whether NCS1-KO mice exhibited altered locomotor and affective behaviors using the OFE tasks (Fig. 5). Female NCS1-KO mice showed no differences compared to female NCS1-WT mice in all measured behaviors. Male NCS1-KO mice also behaved similarly to male NCS1-WT mice in most locomotor activities examined, including total distance moved, vertical entries, and vertical rears (Fig. 5). However, male NCS1-KO mice showed a significant increase in center time and a corresponding decrease in margin time (Fig. 5 C, E, both *p* < 0.01 for male NCS1-WT vs. male NCS1-KO). Although not statistically significant, male NCS1-KO mice also showed a trend towards increased center distance and less margin distance (Fig. 5B,D, both *p* = 0.06 for male NCS1-WT vs. male NCS1-KO), which is expected to co-vary with the changes in time spent in the two zones.

### Affective and cognitive behaviors

#### NCS1-WT and -KO mice show similar anxiety-trait behaviors

**Figure 6:**
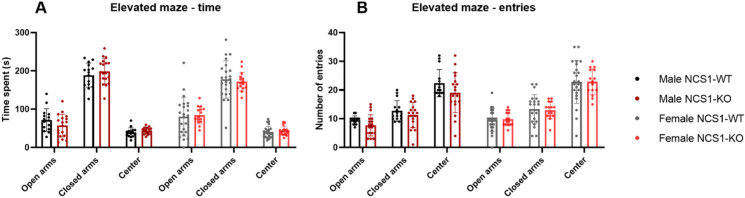
NCS1-WT and -KO mice exhibit no differences in Elevated Plus Maze. (**A**) Both male NCS1-KO and male NCS1-WT mice spent the same amount of time exploring open and closed arms as well as the center (2-way ANOVA, Factor genotype, *p* > 0.99). The same applies to females (2-way ANOVA, Factor genotype, *p* = 0.43). (**B**) No difference in the number of entries to the different sections was observed between the groups [2-way ANOVA, Factor genotype (male), *p* = 0.13; 2-way ANOVA (female), Factor genotype, *p* = 0.99]. Error bars show ± SD. Male NCS1-WT: n = 15, male NCS1-KO: n = 18, female NCS1-WT: n = 15, female NCS1-KO: n = 23.

The elevated plus maze test assesses a rodent's time spent on the open and closed arms. Less time spent on the open arms is considered an anxiogenic-like behavior and can be reversed with common anxiolytic drugs. No difference between NCS1-KO and NCS1-WT mice of both sexes was observed in the time spent on the different segments (Fig. 6). Nevertheless, all groups spent significantly more time in the closed arms of the apparatus (2-way ANOVA, Factor time, *p* < 0.001). There was also no difference in the number of entries to the different segments, suggesting the movement was comparable within groups.

#### Male NCS1-KO mice show impaired performance in the displaced object recognition test (DOR)

**Figure 7:**
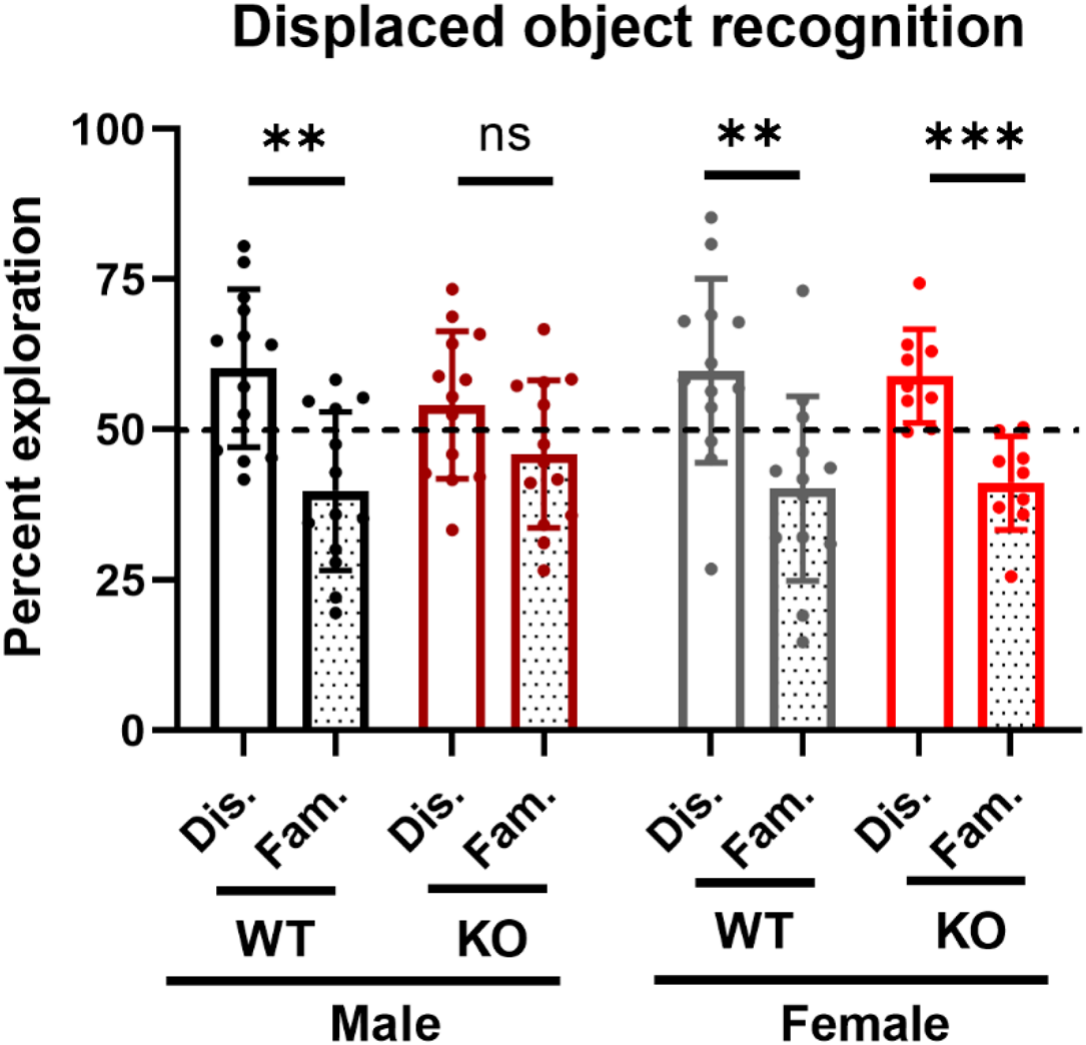
Male NCS1-KO mice show impaired displaced object recognition. (**A**) Both male and female NCS1-WT mice showed normal memory acquisition as determined by the increased preference for the displaced object (two-tailed unpaired t-test, with correction for multiple comparisons, *p* = 0.002 for male NCS1-WT and *p* = 0.006 for female NCS1-WT). Male NCS1-KO mice showed impaired displaced object recognition because they spent similar time exploring the displaced object as the familiar object (t-test, *p* = 0.10). Interestingly, female NCS1-KO mice show intact displaced object recognition (*p* < 0.001). The dotted line denotes 50%, or no preference. Error bars show ± SD. Male NCS1-WT: n = 13, male NCS1-KO: n = 13, female NCS1-WT: n = 12, female NCS1-KO: n = 9.

The DOR test measured short-term spatial memory acquisition. Male NCS1-WT showed a significant preference for the displaced object (Fig. 7, t-test with correction for multiple comparisons, *p* = 0.002). Similar to previous reports^14,33^, we saw that male NCS1-KO mice showed no preference for the displaced object (*p* = 0.1), suggesting that they had impaired memory acquisition. Interestingly, both female NCS1-WT and NCS1-KO mice showed normal displaced object recognition. Their exploration of the displaced object was significantly increased compared to the familiar object (*p* = 0.006 for NCS1-WT and 0.0007 for NCS1-KO).

## Discussion

Here, we investigated the effects of NCS1 deficiency on the peripheral and central nervous systems in both male and female mice. We expected to observe significant alterations in assessments of sensory and motor function between NCS1-WT and NCS1-KO mice. However, only mild changes were detected, primarily in male mice.

### Loss of NCS1 results in mild behavioral differences in male mice

#### Central nervous system

In agreement with previous studies suggesting an important role for NCS1 in learning and cognition^9,14,33–35^, we found that male NCS1-KO mice showed impaired displaced object recognition, whereas female NCS1-KO mice showed normal recognition. Although a previous study reported that NCS1-KO mice showed increased anxiety^34^, another study did not find increased anxiety^14^. Similar to the latter, we did not observe increased anxiety-like behaviors under the conditions examined using the elevated plus maze test. Interestingly, in the open field exploration test, male NCS1-KO mice spent more time in the center area compared to NCS1-WT controls, which may indicate a less anxious phenotype.

#### Peripheral nervous system

The majority of published studies have focused on affective and cognitive differences between NCS1-WT and -KO mice, including anxiety, learning, memory, and social behavior^9,33,34,36,37^. The role that constitutive NCS1 deficiency plays in the peripheral nervous system has, to the best of our knowledge, not been assessed in detail. Several mechanisms have been hypothesized to play an important role in the development of paclitaxel-induced peripheral neuropathy^38^. Because we previously reported the importance of NCS1 for a type of peripheral neuropathy induced by paclitaxel administration^17,18,39,40^, we aimed to find out if constitutive NCS1 deficiency would affect components of the peripheral nervous system. In brief, we found that continuous treatment with paclitaxel led to calpain activation, which in turn degraded NCS1. This downregulation of NCS1, leading to altered intracellular calcium signaling, has been proposed to be a hallmark event in the development of peripheral neuropathy^18^. To assess the whole spectrum of symptoms that peripheral neuropathy presents, various behavioral assays were performed under physiological conditions and in response to several models of acute and inflammatory pain.

For motor performance, grip strength testing and the rotarod assessment did not detect any major differences between NCS1-WT and -KO mice. Locomotor activity was indistinguishable between female NCS1-WT and NCS1-KO mice. In contrast, male NCS1-KO mice exhibited modest changes in some patterns of behavior in the open field exploration task, for example, increased time spent in the center of the open field. Mild differences in this task have been reported previously in NCS1-KO mice^34^. The tail-immersion test did not show any differences between NCS1-WT and NCS1-KO mice across a wide range of sub-threshold and supra threshold temperatures (40–55 °C). Hot and cold plate tests generally did not reveal significant differences in temperature perception. Male NCS1-KO mice showed a decreased escape latency at 50 °C. Why this change in behavior is not observed at more obnoxious temperatures remains unclear. The effect of NCS1 deficiency on temperature sensation may be subtle and, therefore, only observable at less obnoxious temperatures. We suggest the thermal preference test would be useful to investigate potential differences at more subtle changes in temperature^41^. Male NCS1-KO mice also exhibit a decrease in the number of writhes and, therefore, lower visceral pain after acetic acid injection into their peritoneum. Lastly, although Ca^2^-binding proteins have been associated with hearing impairments^42,43^, NCS1 deficiency did not appear to alter hearing function, as indicated by normal ABR and DPOAE responses in NCS1-KO mice.

Unexpectedly, our findings did not show behavioral differences indicating peripheral neuropathy in NCS1-KO mice. Although NCS1 degradation by calpain was shown to play an important role in the development of peripheral neuropathy after paclitaxel administration^18^, NCS1-KO mice did not demonstrate symptoms of peripheral neuropathy. This may be caused by the limitations of a constitutive KO model. Genetic robustness, in which related genes can compensate for the lack of NCS1 during development, may account for the lack of phenotypic differences^44^. For example, when neuroligin-3, a postsynaptic cell-adhesion protein, was knocked out constitutively or during early development, no differences in synaptic transmission were observed^45^. However, knockout of neuroligin-3 during late development resulted in impaired synaptic transmission. Therefore, to replicate the effect of NCS1 deficiency in the peripheral nervous system following paclitaxel administration, NCS1 may need to be knocked down or out in a conditional adult model.

### Male mice exhibit more severe changes in behaviors

Generally, we observed differences between male NCS1-WT and NCS1-KO mice, but not between female NCS1-WT and NCS1-KO mice. A general male bias exists in scientific studies, where single-sex studies of male animals outnumber those of females 5.5 to 1^46^. Similarly, all studies of NCS1-KO mice cited here examined only male mice^14,33,34,37,47,48^. It is often believed that female mammals show more variability than males^46^. Another assumption is that females must be tested across the estrous cycle or that the use of both sexes would reduce statistical power and slow progress^49^. However, it is important to focus on sex-specific differences, which were previously reported to be essential in some mechanisms of pain perception^50,51^.

A possible explanation for why male and female mice show different responses to pain might be due to gonadal hormones. In general, many studies concluded that there is greater nociceptive processing in female rodents^52^. However, new findings show that sex differences in thermal preference of adult mice also occur in the absence of the gonads^53^, suggesting that other factors contribute to differences in thermal sensation. Here, we think it is worth mentioning that the only differences we observed occurred in male mice, posing the question if previous studies' results are highly influenced by the exclusion of female animals in the experiments.

While we recapitulated previous findings that male NCS1-KO mice showed impaired memory acquisition^14,33,44^, unexpectedly, female NCS1-KO mice showed normal memory acquisition. Sex differences in neurodevelopmental and psychiatric disorders are well documented^54–56^. Interestingly, NCS1 deficiency has been associated with autism spectrum disorder (ASD)^14,15^, in which the male-to-female ratio is approximately 3:1^57^. Although the underlying causes of the sex differences in cognition remain to be investigated, this finding supports the suggestion that male NCS1-KO mice may be a good model for ASD.

### Central versus peripheral nervous systems

Another important question is why NCS1 deficiency seems to have a greater impact on the central nervous system than on the peripheral nervous system. It is possible that the expression level of NCS1 is higher in the central nervous system than in peripheral nerves, though this remains to be tested. The function that Ca^2+^ sensors play in the central nervous system appears to be more distinct and diverse than in the peripheral nervous system. Whereas a majority of proteins in the neuronal calcium sensor family are associated with memory and learning, only CaBP5 and Kv channel-interacting proteins (KChIPs) have been associated with peripheral dysfunction, particularly auditory perception and pain, respectively^58^. Nevertheless, the function that NCS1 has in the peripheral nervous system should be studied in detail because it plays an important role in the development of chemotherapy-induced peripheral neuropathy^17–19^.

### Conclusion, limitations, and future directions

Overall, we found that a constitutive loss of NCS1 did not markedly alter sensory or motor performance in mice, and NCS1-KO mice did not exhibit peripheral neuropathy. Only mild changes in sensory perception were observed, and only in male NCS1-KO mice. Increasing the number of mice in each pain assay cohort will also help to discover subtle baseline differences. A conditional KO of NCS1 in the peripheral nervous system becomes more relevant to understanding the function that post-natal and disease-related changes in NCS1 expression play for signal transduction and the health of sensory and motor neurons in adult mice. To assess the biochemical basis for the observed differences, an in-depth comparison of intracellular calcium signaling is needed using dorsal root ganglia extracted from NCS1-WT and NCS1-KO mice. Furthermore, biochemical assays that investigate calpain activity and its targets are needed to assess underlying mechanisms, especially as calpain targets are hypothesized to play a crucial role in acute and chronic pain. Our findings also suggest that sex-dependent factors must also be considered to identify possible underlying mechanisms of pain perception.

## Materials and methods

### Animal use and treatment (Ehrlich lab)

NCS1-KO mouse strain on C57Bl/6 J background was a gift from O. Pongs (University of the Saarland, Homburg). Details about how this strain were generated were previously published^37,59^. Briefly, the strain was generated by gene targeting and Cre-Lox recombination that resulted in a loss of 4 translated exons of NCS1, and hence a complete loss of NCS1 protein expression in all tissues. This study was carried out in accordance with the recommendations in the U.S. National Institutes of Health Guide for the Care and Use of Laboratory Animals. The protocol was approved by the Institutional Animal Care and Use Committee at Yale University. All efforts were made to minimize suffering. Mice were maintained on a 12:12-h light/dark cycle (7:00 a.m. on/7:00 p.m. off) with food and water provided ad libitum before experimental procedures. All animal experiments were performed during the light cycle. These mice were used to generate data for the displaced object recognition, accelerated rotarod, and hearing tests.

### Animal use and treatment (Bilsky lab)

The mice were obtained from the Ehrlich lab, sent to the Bilsky lab, and used to examine open field exploration, constant speed rotarod, hot/cold plate, tail-flick, Acetic Acid writhing, Carrageenan injection, von Frey, Hargreaves tests^31^. The protocol was approved by the Institutional Animal Care and Use Committee at the University of New England, and detailed behavioral protocols are provided in the sections below. All efforts were made to minimize the number of animals utilized as well as pain and suffering. Mice experiencing more prolonged inflammatory and post-surgical pain assays were euthanized immediately after the last time-point. Mice were maintained on a 12:12-h light/dark cycle (7:00 a.m. on/7:00 p.m. off) with food and water provided ad libitum before experimental procedures. All animal experiments were performed during the light phase.

### Cold/hot plate

A slightly modified version of the technique of Eddy and Leimbach (1953)^60^ was used. Animals were placed on a metal surface maintained at various temperatures to obtain the animals' latency to escape (s). Cold plate was tested at 10, 5, and 2 °C, and hot plate was tested at 50, 52, and 55 °C. We set cut-off times (90 s for 10 and 5 °C, 60 s for 2 °C, and 30 s 50, 52, and 55 °C) to prevent tissue damage in animals not responding to the stimulus. Locomotion of the animal on the plate was constrained by a Plexiglas tube. Latency to respond to the stimulus was measured to the nearest 0.1 s. Animals remained on the plate until they performed either of two behaviors regarded as indicative of nociception: hind paw lick or hind paw shake/flutter^61^.

### Tail flick

The tail-flick test assessed the mice's spinal reflex to respond to various temperatures of warm water (40, 43, 46, 49, 52, and 55 °C). The latency to the first sign of a rapid tail flick was taken as the behavioral endpoint^62^. Each mouse was tested for their response latency by immersing the distal third of the tail into the water and recording the time to respond. Various cut-off times were used to avoid tissue damage when the animal did not respond to the stimulus (15 s for 40–49 °C and 10 s for 52–55 °C).

### Acetic acid writhing

In this assay of chemical nociception^63–65^, a noxious substance (0.56% glacial acetic acid (AA)) was injected into the peritoneal cavity, wherein it activates nociceptors directly or produces inflammation of visceral (subdiaphragmatic organs) and subcutaneous (muscle wall) tissues. 0.56% AA was injected intraperitoneally (i.p.) at a volume of 10 ml/kg, bodyweight into the mice. This injection produces a characteristic ‘writhing' response that was be quantified by trained observers. Immediately following injection, mice were placed in clear Plexiglass tubes with a filter paper bottom and were videotaped and observed for 20 min. The total number of writhes (lengthwise stretches of torso with a concomitant concave arching of the back) was counted and totaled.

### Carrageenan injection

Inflammatory hyperalgesia was induced by the injection of 2% carrageenan (Sigma Aldrich) at a volume of 0.50 ml into the plantar surface of the mouse's left hind paw. Behavioral testing occurred in the von Frey test pre-injection and at 3.5hrs post-injection.

### Plantar incision surgery

Prior to surgery, all mice were baselined for hind paw thresholds in the von Frey (tactile) and Hargreaves (thermal) assays^31^. A longitudinal incision through the plantaris muscle of the hind paw was used to induce post-surgical pain^66^. Briefly, mice were anesthetized by inhalation of isoflurane (induction, 5%; surgery 2%). A 0.5–0.75 cm longitudinal incision was carefully made with a number 11 blade, through skin and fascia of the plantar aspect of the foot. The plantaris muscle was elevated and incised longitudinally with a number 11 blade (leaving intact the muscle origin and insertion). A #6-0 nylon suture was used to suture the skin, and Neosporin was then applied topically to the wound site to prevent infection. After surgery, mice were allowed to recover in their home cages and were re-tested for tactile and thermal latencies 24, 48, and 72 h post-surgery.

### Von Frey test

Tactile allodynia was quantified by measuring the hind paw withdrawal threshold to von Frey filament stimulation, using the up-down method previously reported^67^. Animals were placed in a suspended plastic chamber with a wire mesh platform and allowed to habituate. Tactile thresholds were measured by probing the left hind paw's plantar surface with a series of calibrated von Frey filaments having bending forces of (2.44, 2.83, 3.22, 3.61, 4.08, 4.31, and 4.56). Withdrawal thresholds were determined by sequentially increasing and decreasing stimulus intensity ("up and down" method, starting filament strength = 3.61), analyzed by using a Dixon nonparametric test, and expressed as the paw withdrawal threshold in gram force values^68^.

### Hargreaves (thermal latency) test

For assessing thermal withdrawal latencies, mice were acclimatized for ~ 60 min to Plexiglas holding chambers that rest on a glass surface maintained at room temperature. The surface was cleaned of urine and feces before assessments. Thermal nociceptive thresholds were determined similarly to the methods described by Hargreaves et al.^31^. Briefly, a radiant heat source (Ugo Basile) was focused through the glass surface onto the left hind paw's plantar surface. Upon paw withdrawal, the heat stimulus was automatically deactivated, and the latency to withdraw is recorded to the nearest 0.1 s. The intensity of the light stimulus was set at 28 to obtain baseline latencies around 15 s. The test was terminated if no response occurred at 30 s to prevent tissue damage.

### Open-field exploration

The open-field test measured general levels of arousal and locomotor activity using an automated open-field activity monitoring system (Coulbourn Instruments with TruScan software). The open-field test is a fully automated assay that quantifies many different aspects and patterns of movement, including total distance traveled, time spent moving, and vertical rearing. The software also calculated the time spent in the middle portions of the open field versus the outer zones alongside the outside walls, variables that can be used as one measure of general anxiety levels. Animals were placed into an open field in a brightly lit room. The open-field chambers are surrounded by two sensor rings that have arrays of infrared beams that feed data into the computer program for analysis. The TruScan software interprets the beam breaks to calculate a wide variety of movement variables and patterns of activity, with a temporal resolution of 100 ms. A 60 min session was conducted, and data were graphed as the average total distance the mouse traveled in the test session.

### Rotarod

The apparatus (IITC Series 8 RotaRod) consists of a horizontal rod, capable of rotating at various speeds, positioned above the floor pan below. The rod's length is divided into individual compartments allowing for the running of multiple animals at a time. The rod was set to rotate at a constant rate of 15 RPM's and testing began once the mouse was positioned on the rod. The test was concluded when one of the following events occurred; the mouse fell off the rod, the mouse made one complete revolution on the rod without walking, or the mouse reached the cut-off of 180 s. Testing occurred on two consecutive days, each day consisting of 4 rotarod trials. For the accelerated rotarod, the speed was increased from 0 revolutions per minute (rpm) to 40 rpm over 300 s with a constant acceleration rate. Testing was concluded when the mouse fell off the rod, or the mouse reached the cut-off of 300 s. 4 consequent trials were tested on one day.

### Grip strength

When a mouse is pulled by their tail, they instinctively grab what is located directly in front of them. The assessment of grip strength is a measure of the strength behind that grasp. To measure this phenotype, a grip strength meter (Ugo Basile, Cat. No. 47200) was used. The animal was placed facing a grasping bar, fitted with a force sensor, connected to a control unit. Once positioned, the rodent was pulled backward at a consistent rate/force by a trained experimenter, and the animal grasped onto the bar in front of them. At the point where the force of the experimenter becomes too great, the animal lost its grip, and the control unit displayed the force (grams) at which the animal released the bar. This test was conducted for all animals in the study, then replicated two additional times to obtain three readings per animal that were then averaged together for one grip strength measurement.

### Elevated plus maze

This test is derived from the innate aversion of rodents to open spaces, whereby animals that spend less time exploring open arms are thought to behave more anxiously (Pellow et al. 1985). The Elevated Plus Maze (EPM) is in the shape of a +, with four arms (length: 45 cm, width: 12 cm, height from floor: 70 cm) and a central region (12 × 12 cm^2^). Two arms are dark and enclosed on three sides by walls (45 cm), and the other two arms are open. A source of dim light (~ 70 lx) and a video camera are placed above the center of the maze. The test is started by placing the mouse in the central region and is concluded after 5 min has passed. The mouse's movements (number of entries into the open arms vs. closed arms and total time spent (s) in the closed vs. open arms) are watched and quantified by trained observers. The apparatus is wiped clean with 70% ethanol and dried between trials.

### Auditory brainstem responses (ABRs) and distortion product otoacoustic emissions (DPOAEs)

Hearing sensitivity of NCS1-KO and NCS1-WT mice at 2.5 months of age of either sex was measured using ABRs, which represent synchronized electrical activity in the auditory nerve and ascending central auditory pathways in response to acoustic stimuli, and DPOAEs, which are sounds generated by outer hair cells in the cochlea in response to a two-tone stimulus. As described previously^69^, measurements were carried out within a sound-attenuating booth (Industrial Acoustics Corp., Bronx, NY, USA). Mice were anesthetized with chloral hydrate (480 mg/kg i.p), and placed onto a heating pad, to maintain body temperature at 37 °C. The acoustic stimuli for ABR and DPOAE were produced, and the responses recorded using a TDT System 3 (Tucker-Davis Technologies, Inc., Alachua, FL, USA) controlled by BioSigRP (TDT), a digital signal processing software.

ABRs were measured as previously described^69^ by placing subdermal needle electrodes at the vertex (active, noninverting), the right infra-auricular mastoid region (reference, inverting), and the left neck region (ground). ABRs were elicited with pure tone pips presented free field via a speaker (EC1 Electrostatic Speaker, TDT) positioned 10 cm from the vertex. Symmetrically shaped tone bursts were 3 ms long (1 ms raised cosine on/off ramps and 1 ms plateau) and were delivered at a rate of approximately 21 per second. Stimuli were presented at frequencies between 2 and 32 kHz in half-octave steps and in 5 dB decrements of sound intensity from 90 dB SPL. Differentially recorded scalp potentials were bandpass filtered between 0.05 and 3 kHz over a 15-ms epoch. A total of 400 responses were averaged for each waveform for each stimulus condition. The ABR threshold was defined as the lowest sound intensity capable of evoking a reproducible, visually detectable response. Amplitudes (µV) and latencies (ms) of the first ABR wave (wave I) were determined at 16 kHz. The most sensitive frequency range of hearing in mice is 11.3–22.6 kHz, and 16 kHz is half octave in-between, so was therefore chosen for analysis. The analysis was carried out offline in BioSigRP on traces with visible peaks by setting cursors at the maxima and minima (trough) of the peaks. Latency was determined as the time from the onset of the stimulus to the peak, while amplitude was measured by taking the mean of the ∆V of the upward and downward slopes of the peak.

DPOAEs were measured by inserting a microphone probe (ER-10B + Microphone System, Etymotic Research, Inc., Elk Grove Village, IL, USA) into the external ear canal of the anesthetized mouse, with two speakers (MF1 Multi-Field Magnetic Speakers, TDT) connected to the probe via tubing. Two simultaneous continuous pure tones (f_1_ and f_2_) that have a frequency ratio of 1.2 (f_2_/f_1_) and equal sound level were delivered at center frequencies of 8, 12, 16, 24, and 32 kHz with sound levels from 80 to 20 dB SPL in 10 dB decrements. Stimulus duration was 83.88 ms at a repetition rate of 11.92 per second. The acquired DPOAE responses were averaged 100 times. The DPOAE of interest was at 2f_1_-f_2_, the largest and most prominent DPOAE. The DPOAE threshold was defined as the lowest sound intensity capable of evoking a visually detectable 2f_1_-f_2_ signal above the noise floor. DPOAE amplitudes (dB SPL) were determined at the f_2_ frequency of 17.44 kHz across all the sound intensity levels above the threshold. The analysis was carried out offline in BioSigRP by setting cursors at the peak of the 2f_1_-f_2_ signals.

### Displaced object recognition

Behavioral experiments were adapted from a published protocol^70^. Data were analyzed blinded to the genotype. The experimental arena was a 35 × 70 × 35 cm opaque, white Plexiglas chamber. The arena was covered with ~ 1 cm of standard corn cob bedding. After each mouse, feces were removed, and the bedding was shaken to distribute odor cues equally. A camera was mounted 100 cm above the arena to record the test sessions. The test was conducted during the mice's light phase under low light conditions (45 lx). 1 h before testing, mice were brought up and allowed to habituate to the testing room. Pairs of 50-mL Falcon tubes filled with corn cob bedding were taped cap-down to pre-determined positions in the arena. They were selected specifically because mice were unable to climb onto the pointed ends of the tubes. During the familiarization phase, each mouse was first allowed to explore the arena where the two Falcon tubes were placed in symmetrical locations for 5 min before being taken out and returned to its home cage. 2 h later, the mouse was returned to the arena for another 5 min, with 1 tube remained in the same position and 1 tube moved to a different position. The positions of the tubes were counterbalanced. After each mouse, the tubes were sprayed with 70% ethanol, wiped with tissue paper, then sprayed with water and wiped dry to remove odor cues. The camera footage was then analyzed for bouts of interactions with the tubes. Sniffing and biting were considered to be interaction. Casual touching of the tubes in passing, or leaning onto the tubes to look around were not counted. The percent exploration for the displaced object was calculated as 100* (time spent with displaced object)/(total time spent with both displaced and familiar objects). The percent exploration for the familiar object was similarly calculated.

### Statistical analyses

Data management and calculations were performed using PRISM Statistical Software 8 (GraphPad Software, Inc, California). We used t-test or mixed analyses of variance (ANOVA) followed by multiple comparison tests with Sidak’s correction to compare genotypes individually. Factor dependent *p* values are reported in the figure legend. Generally, a *p* value < 0.05 was considered to be statistically significant and the following notations were used in all figures: * for *p* < 0.05, ** for *p* < 0.01, *** for *p* < 0.001, and **** for *p* < 0.0001. For all bar graphs, error bars show standard deviation (SD). XY graphs show the standard error of the mean (SEM).

### Ethics approval

The animal protocol followed the recommendations in the U.S. National Institutes of Health Guide for the Care and Use of Laboratory Animals, and was approved by the Institutional Animal Care and Use Committee at Yale University and the University of New England.

### Consent for publication

All authors consented to the publication of the manuscript.

## Data Availability

The datasets used and/or analyzed during the current study are available from the corresponding author upon reasonable request.

## Abbreviations

ANOVA: Analyses of variance
ABR: Auditory brainstem response
ASD: Autism spectrum disorder
DOR: Displaced object recognition
DPOAE: Distortion product otoacoustic emission
IHCs: Inner hair cells
NCS1: Neuronal calcium sensor 1
OHCs: Outer hair cells
OFE: Open-field exploration
